# Inhibition of P2X7 receptor ameliorates transient global cerebral ischemia/reperfusion injury via modulating inflammatory responses in the rat hippocampus

**DOI:** 10.1186/1742-2094-9-69

**Published:** 2012-04-18

**Authors:** Ketan Chu, Bo Yin, Jingye Wang, Guoping Peng, Hui Liang, Ziqi Xu, Yue Du, Marong Fang, Qiang Xia, Benyan Luo

**Affiliations:** 1 Department of Neurology, First Affiliated Hospital, Zhejiang University School of Medicine, 76# Qingchun Road, Hangzhou, 310003, China; 2 Brain Medical Center, First Affiliated Hospital, Zhejiang University School of Medicine, Hangzhou, China; 3 Department of Physiology, Zhejiang University School of Medicine, 388# Yuhangtang Road, Hangzhou, 310058, China; 4 Key Laboratory of Medical Neurobiology, Ministry of Health, Hangzhou, China; 5 Department of Neurology, First Affiliated Hospital, Anhui Medical University, Hefei, China; 6 Institute of Anatomy and Cell Biology, Medical College, Zhejiang University, Hangzhou, China

**Keywords:** P2X7 receptor, Ischemia/reperfusion injury, Inflammation, Microglia, Cytokine

## Abstract

**Background:**

Neuroinflammation plays an important role in cerebral ischemia/reperfusion (I/R) injury. The P2X7 receptor (P2X7R) has been reported to be involved in the inflammatory response of many central nervous system diseases. However, the role of P2X7Rs in transient global cerebral I/R injury remains unclear. The purpose of this study is to determine the effects of inhibiting the P2X7R in a rat model of transient global cerebral I/R injury, and then to explore the association between the P2X7R and neuroinflammation after transient global cerebral I/R injury.

**Methods:**

Immediately after infusion with the P2X7R antagonists Brilliant blue G (BBG), adenosine 5′-triphosphate-2′,3′-dialdehyde (OxATP) or A-438079, 20 minutes of transient global cerebral I/R was induced using the four-vessel occlusion (4-VO) method in rats. Survival rate was calculated, neuronal death in the hippocampal CA1 region was observed using H & E staining, and DNA cleavage was observed by deoxynucleotidyl transferase-mediated UTP nick end labeling TUNEL). In addition, behavioral deficits were measured using the Morris water maze, and RT-PCR and immunohistochemical staining were performed to measure the expression of IL-1β, TNF-α and IL-6, and to identify activated microglia and astrocytes.

**Results:**

The P2X7R antagonists protected against transient global cerebral I/R injury in a dosage-dependent manner. A high dosage of BBG (10 μg) and A-0438079 (3 μg), and a low dosage of OxATP (1 μg) significantly increased survival rates, reduced I/R-induced learning memory deficit, and reduced I/R-induced neuronal death, DNA cleavage, and glial activation and inflammatory cytokine overexpression in the hippocampus.

**Conclusions:**

Our study indicates that inhibiting P2X7Rs protects against transient global cerebral I/R injury by reducing the I/R-induced inflammatory response, which suggests inhibition of P2X7Rs may be a promising therapeutic strategy for clinical treatment of transient global cerebral I/R injury.

## Background

Transient global cerebral ischemia is one of the major complications of clinical emergencies such as cardiac arrest, drowning or severe systemic hypotension during a surgical procedure. Currently, the most adequate treatment for these patients is re-establishing perfusion of the brain as soon as possible. However, reperfusion may paradoxically exacerbate brain injury, which is called cerebral ischemia/reperfusion(I/R) injury [1]. Therefore, efforts need be made that not only preserve cerebral blood flow, but also prevent the actual mechanisms that trigger brain damage after I/R injury [2].

Neuroinflammation, which is characterized by microglial and astroglial activation, as well as the release of cytotoxic agents (cytokines, matrix metalloproteinases, nitric oxide and reactive oxygen species) can be triggered by cerebral I/R injury, which can contribute to blood–brain barrier disruption and delayed neuronal death [3]. Subsequently, these damaged cells release more toxic mediators, which in turn activate more immune cells. Thus, prolonged inflammation caused by this vicious circle exacerbates brain damage. Taken together, anti-inflammation therapy may become a promising therapeutic strategy for the treatment of cerebral I/R injury [3,4].

The P2X7 receptor(P2X7R), a purinergic receptor, was first discovered in macrophages. In the central nervous system (CNS), the P2X7R is predominantly expressed in microglia which are the resident macrophages of the brain [5]. The P2X7R can be activated by high concentrations of ATP. Stimulating the P2X7R leads to microglial activation, reactive oxygen species production and increased secretion of pro-inflammatory cytokines such as IL-1ß, TNF-α and IL-6 [6,7]. Recently, the P2X7R has been reported to be involved in neuroinflammation in many CNS diseases including Alzheimer’s disease (AD), epilepsy, spinal cord injury and multiple sclerosis, and treatment with P2X7R antagonists reduces experimentally induced neuroinflammation in animal models of such diseases [6,8-12].

The P2X7R has also been reported to participate in cerebral ischemic injury. *In vitro* and *in vivo* studies have shown that inhibition of P2X7Rs reduced oxygen and glucose deprivation-induced oligodendrocyte death [13] as well as infarct volume after transient middle cerebral artery occlusion(MCAO) injury [14,15]. However, ischemic injury exacerbation by P2X7R antagonists has also been reported [16,17]. To date, the contribution of the P2X7R to cerebral ischemic injury remains an issue, and whether inhibition of P2X7R has beneficial or harmful effects in global cerebral I/R injury has not been studied. We, therefore, designed experiments using two widely used P2X7R antagonists, Brilliant blue G (BBG) and adenosine 5′-triphosphate-2′,3′-dialdehyde (OxATP) [6], and the selective P2X7R antagonist A-438079 [18] to investigate the role of P2X7R in a rat model of transient global cerebral I/R injury. We also explored the association between the P2X7R and neuroinflammation after transient global cerebral I/R injury.

## Methods

### Animals and surgical procedures

Male Sprague–Dawley rats weighing 260–320 g were provided by the Animal Center of Zhejiang University. All procedures used in this study were carried out according to the guidelines of the NIH Guide for the Care and Use of Laboratory Animals and have been approved by the Ethics Committee for the Use of Experimental Animals in Zhejiang University.

Twenty minutes of global cerebral ischemia was induced by the four-vessel occlusion (4-VO) method with slight modification, as established by Pulsinelli [19], and routinely used in our laboratory [20,21]. Briefly, anesthesia was induced with 4% (w/v) choral hydrate (400 mg/kg, intraperitoneally (i.p.)), then the bilateral common carotid arteries (CCAs) were freed and both vertebral arteries were permanently electrocauterized. Rats were allowed to recover for 24 hours after closing the surgical incisions.

On the following day (+0D), anesthesia was applied, the surgical incision in the neck was opened and both CCAs were occluded with aneurysm clips to induce global cerebral ischemia. The clips were removed for reperfusion. Rectal temperature was maintained at 36.5 to 37.5°C throughout the procedures. Cerebral blood flow (CBF) before and after clamping the bilateral CCAs was monitored using a laser Doppler blood flow monitor(PeriFlux System5000, Perimed, Sweden), and rats with a decrease in CBF of less than 80% were excluded [22].

### Drug administration and experimental groups

To study the neuron survival rate in the hippocampal CA1 region after transient global cerebral I/R injury, rats were divided into eight groups: sham group (sham operated), saline group (I/R + saline 2μL intracerebroventricular (i.c.v.), BBG (Sigma, St. Louis, MO) 1 μg, 5 μg, and 10 μg groups (I/R + BBG 1 μg/5 μg/10 μgi.c.v.), OxATP (Sigma, St. Louis, MO) 1 μg, 5 μg, and10 μg groups (I/R + OxATP 1 μg/5 μg/10 μgi.c.v.) and A-438079 (Tocris Bioscience, Ellisville, MO) 0.03 μg, 0.3 μg, and 3 μg groups (I/R + A-438079 0.03 μg/0.3 μg/3 μgi.c.v.). For the other studies, rats were divided into five groups: sham group, saline group, BBG 10 μg group(I/R + BBG 10 μg i.c.v.), OxATP 1 μg group (I/R + OxATP 1 μg i.c.v.) and A-438079 3 μg group (I/R + A-438079 3 μg i.c.v.).

Drug or saline was injected into the right cerebral ventricle (AP −0.92 mm, ML 1.5, DV 3.5 mm) using a microinjector. Drugs were administered into the lateral cerebral ventricle 10 minutes prior to global cerebral ischemia with a total volume of 2 μL at a speed of 0.5μL/minute.

### Sample preparation

After three (+3D) or seven days (+7D) of reperfusion, rats were anesthetized and perfused intracardially with saline, followed by 4%(w/v) paraformaldehyde in 0.1 mol/L PBS, pH = 7.4. Brains were removed and fixed overnight in 4%(w/v) paraformaldehyde. Brains were embedded in paraffin, and cut into 4 μm coronal sections at the level of the bregma for H&E staining or immunohistochemistry.

### Morris water maze

At +8D, spatial learning and memory were tested using the Morris water maze [23] which was a circular tank of 120 cm in diameter and 50 cm in height. The tank was filled to a depth of 30 cm with water at 25 ± 1°C. The water was made opaque by adding Chinese ink. The maze was divided into four equal quadrants. The trials were performed according to Vorhees’ method [24].

Spatial acquisition: All rats received a training trial consisting of daily sessions of four consecutive trials for five days. The hidden platform (diameter 10 cm, 1.5 cm below the water surface) was positioned in the middle of the southwest (SW) quadrant for all rats. The rats were released into the tank facing the maze wall at north (N), west (W), south (S), or east (E) quadrants in a predetermined pseudorandom order. A trial was terminated as soon as the rat found the platform; if the rat did not succeed within 120 seconds, it was guided onto the platform with a stick. The rat was allowed to stay on the platform for 20 seconds before being removed.

Probe trial: Immediately after the final training trial, the platform was removed. Rats were released into the pool at NE position and allowed to swim freely for 2 minutes. The time needed to find the platform (escape latency) in the training trials and time spent in the SW quadrant in the probe trial were recorded. The mean value of four escape latencies in the daily four training trials was taken as the escape latency for the rat. Values from eight rats in the same group were averaged to generate a mean escape latency for that day. Brains of rats in the behavioral study were removed after two weeks of reperfusion and stained with H&E, and the surviving neurons were counted.

### Immunohistochemistry

Immunohistochemistry was performed according to the protocol of Wang *et al.*[20]. Briefly, sections were prepared at +3D using the same method mentioned in ‘sample preparation’. Sections were dewaxed with xylene and dehydrated by ethanol at graded concentrations and distilled water. Sections were then incubated for 10 minutes in 3%(v/v) hydrogen peroxide to block endogenous peroxidase activity. High-temperature antigen retrieval was performed in 0.01 M citrate buffer, pH 6.0, for 20 minutes. The brain sections were incubated overnight at 4°C with rabbit anti-Iba1 (ionized calcium-binding adaptor molecule 1), immunoglobulin G(IgG) (diluted 1:500, Wako, Osaka, Japan) or mouse anti-GFAP (glial fibrillary acidic protein) IgG (diluted 1:50, Millipore, Bedford, MA, USA) in PBS containing 0.3%(v/v) Triton X-100, followed by incubation with EnVision (Zymed, San Francisco, CA, USA) solution at 37°C for 30 minutes. Finally, sections were incubated with peroxidase substrate diaminobenzidine until a desired staining intensity developed, followed by slight counterstaining with hematoxylin, dehydration, and cover-slipping with permount. Between incubations, the tissue was washed with PBS three times for 10 minutes each.

*In situ* labeling of DNA fragmentation (terminal deoxynucleotidyl transferase-mediated UTP nick end labeling (TUNEL)) was preformed with an *in situ* cell death detection kit (Roche, Mannheim, Germany) according to the manufacturer’s instructions.

### RT-PCR

Hippocampi were rapidly isolated at +3D and total RNA was extracted with TRIzol reagent(Invitrogen, Carlsbad, CA, USA) following the manufacturer’s protocol. RNA (1 μg) was reverse transcribed using random primers and M-MLV reverse transcriptase (Promega, Madison, WI, USA) . The resulting cDNA amplification was performed using the 7500 Fast Real-Time System (Applied Biosystems, Foster City, CA, USA) in conjunction with the SYBR Premix Ex Taq^TM^ II kit (Takara, Japan). The endogenous housekeeping gene, GAPDH (glyceraldehyde-3-phosphate dehydrogenase), was used as a loading control to normalize target gene expression. Each sample was analyzed in triplicate. Relative expression levels of the target cDNAs were quantified by the 2-ΔΔCts method. The target genes and the specific primers are shown in Table 1.

**Table 1:** Details of primers (forward & reverse) for different genes used for RT-PCR analysis

**Gene**	**Primer**	**Sequences**
IL-1ß	forward	5′-GCTGTGGCAGCTACCTATGTCTTG-3′
	reverse	5′-AGGTCGTCATCATCCCACGAG-3′
TNF-α	forward	5′-ATACACTGGCCCGAGGCAAC-3′
	reverse	5′-CCACATCTCGGATCATGCTTTC-3′
IL-6	forward	5′-CCACTTCACAAGTCGGAGGCTTA-3′
	reverse	5′-GTGCATCATCGCTGTTCATACAATC-3′

### Quantitative analysis

Neuronal damage and apoptosis were quantified by counting the number of surviving neurons or immunopositive neurons for TUNEL in the hippocampal CA1 layer. Photomicrographs of the CA1 region were taken using a digital camera (Olympus DP20, Tokyo, Japan) connected to an inverted microscope (Olympus BX51, Tokyo, Japan). The numbers of surviving/TUNEL-positive neurons and total neurons in the hippocampal CA1 layer per 1 mm length were counted and analyzed by blinded investigators. The severity of neuronal damage/apoptosis was evaluated in the form of neuron survival rate/TUNEL-positive neuron rate which was calculated by the number of surviving or TUNEL-positive neurons/the number of total neurons.

To quantify glial activation, we measured the value of the integrated optical density (IOD) for Immunohistochemistry images from the CA1 region stained with antibodies against Iba-1 or GFAP using Image-Pro Plus 6.0 (Media Cybernetics, Bethesda, MD, USA). Pictures were taken using the same method as mentioned above. Three high power (400×) images were randomly selected for each animal, and the mean IOD of these three fields was considered as the IOD of the animal.

### Statistical analysis

Values are expressed as the mean ± S.E.M. The Kaplan-Meier method was used in the analysis for survival and data were compared using the log-rank test (GraphPad Prism 5). For analysis of escape latency in the behavioral study two-way analysis of variance(ANOVA) was performed. Other data were subjected to one-way ANOVA and post-hoc comparisons were carried out using Bonferroni’s test. Values of *P* < 0.05 were considered significant.

## Results

### Inhibition of the P2X7R reduces I/R-induced neuron death and neuronal DNA cleavage

**Figure 1:**
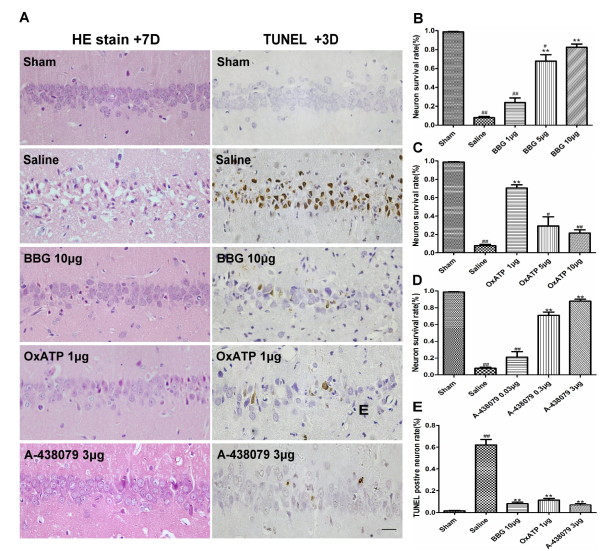
**Effect of BBG, OxATP and A-438079 on I/R-induced neuronal damage in the hippocampal CA1 region. (A)** Representative H&E staining of hippocampal CA1 neurons from the sham group, saline group, BBG 10 μg group, OxATP 1 μg and A-438079 3 μg groups at the seventh day (+7D) (left) and representative TUNEL of hippocampal CA1 neurons from the sham group, saline group, BBG 10 μg group, OxATP 1 μg and A-438079 3 μggroups at the third day (+3D). (**B)** Neuron survival rate in the hippocampal CA1 region in BBG 1 μg, 5 μg and 10 μg groups at +7D. (**C)** Neuronal survival rate in the hippocampal CA1 region in OxATP 1 μg, 5 μg and 10 μg groups at +7D. (**D)** Neuronal survival rate in the hippocampal CA1 region in the A-438079 0.03 μg, 0.3 μg and 3 μg groups at +7D. (**E**) The percentage of TUNEL-positive neurons in the hippocampal CA1 region in the sham, saline, BBG 10 μg, OxATP 1 μg and A-438079 3 μg groups at +3D. Values are expressed as the mean ± S.E.M. All photomicrographs are × 400. Scale bar: 10 μm. **P < 0.01 versus saline group; * *P* < 0.05 versus saline group; ##*P* < 0.01 versus sham group; # *P* < 0.05 versus sham group. (n = 6 in all the groups). BBG, Brilliant blue G; I/R, ischemia/reperfusion; OxATP, adenosine 5′-triphosphate-2′,3′-dialdehyde; S.E.M., standard error of the mean; TUNEL, deoxynucleotidyl transferase-mediated UTP nick end labeling.

To address the potential that inhibiting P2X7R prevents transient global cerebral I/R injury, neuronal survival was observed at +3D and +7D. Morphologically, most neuronal death occurred within 72 hours (data not shown). Only 10.8 ± 4.2% and 7.8 ± 3.3% of the hippocampal CA1 neurons survived in the saline group at +3D and +7D, respectively. All three P2X7R antagonists protected against transient global cerebral I/R injury in a dose-dependent manner. BBG 5 μg and BBG 10 μg treatment significantly increased the neuron survival rate when compared to BBG 1 μg and saline; however, the BBG 10 μg was more effective (Figure 1A,B). Rats receiving 1 μgOxATP had significantly more surviving hippocampal CA1 neurons than those receiving other doses or saline (Figure 1A,C). Neuronal survival in A-438079 treated animals was similar to survival rates in those treated with BBG and A-438079 0.3 μg and A-438079 3 μg treated animals, with significantly more surviving hippocampal CA1 neurons when compared to the A-438079 0.03 μg and saline groups (Figure 1A,D). Rats from the A-438079 3 μg group had higher neuron survival rates than those from the OxATP 1 μg group (87.8 ± 6.0% versus70.4 ± 8.6%). The neuron survival rate in the BBG 10 μg and A-438079 3 μg group did not differ from each other at +7D.

Neuronal DNA cleavage induced by transient global cerebral I/R injury was assessed by TUNEL at +3D. Most of the morphologically damaged CA1 neurons were also positive for TUNEL. The TUNEL-positive neuron rate was 62 ± 10.1% in the saline group. The number of TUNEL-positive CA1 neurons at +3D of reperfusion was reduced almost completely in the BBG 10 μg (8 ± 2.8%), OxATP 1 μg (11.3 ± 3.3%) and A-438079 3 μg (6.8 ± 2.4%) groups. There was no significant difference among the BBG 10 μg, OxATP 1 μg, A-438079 3 μg groups and the sham group (Figure van A, E).

### Inhibition of P2X7Rs increases survival rate

**Figure 2:**
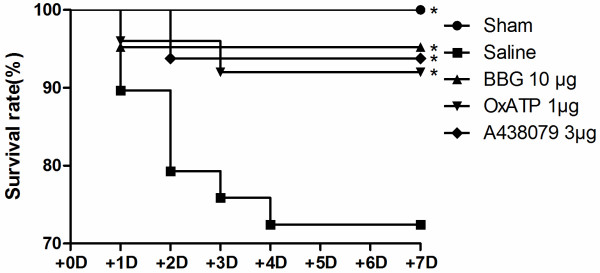
**Survival rate at seven days after transient global cerebral I/R injury.** Values are expressed as a percentage. * *P* < 0.05 versus saline group (n = 20, 28, 20, 22, 16 in sham, saline, BBG 10 μg, OxATP 1 μg and A-438079 3 μg groups, respectively). BBG, Brilliant blue G; I/R, ischemia/reperfusion; OxATP, adenosine 5′-triphosphate-2′,3′-dialdehyde.

The survival rates of rats during the seven days after I/R injury was significantly reduced in the BBG 10 μg (95.2%), OxATP 1 μg(92.0%) and A-438079 3 μg (93.8%) treated groups when compared to the saline group(72.4%). No significant difference was observed among the BBG 10 μg, OxATP 1 μg and A-438079 3 μg groups (Figure 2).

### Inhibition of P2X7Rs improves I/R-induced behavioral deficits

**Figure 3:**
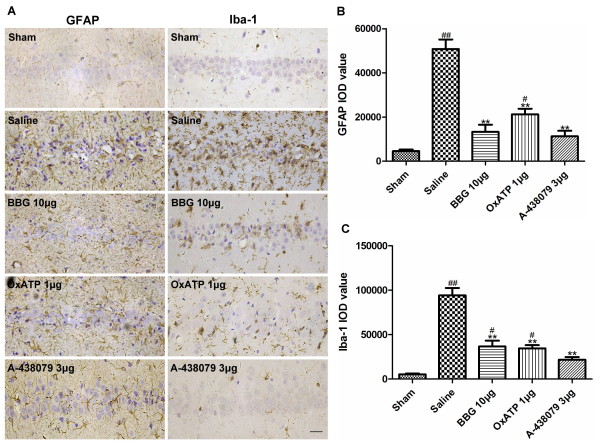
**Effect of BBG, OxATP and A-438079 on spatial learning and memory in the water maze. (A)** Escape latency to find the platform during the five days of thetraining trial from the eighth day (+8D) to the twelfth day (+12D) after reperfusion. (**B)** Time spent during the probe trial in the quadrant where the platform had been located. Values are expressed as the mean ± S.E.M. *P < 0.05 versus the corresponding value of thesaline group on the same day; **P < 0.01 versus the corresponding value of the saline group on the same day; #*P* < 0.05 versus the corresponding value of the sham group on the same day; ##*P* < 0.01 versus the corresponding value of the sham group on the same day; + *P* < 0.05 versus the corresponding value of the OxATP 1 μg group on the same day; ++*P* < 0.01 versus the corresponding value of the OxATP 1 μg group on the same day (n = 15, 14, 13, 13, 10 in sham, saline, BBG 10 μg, OxATP 1 μg, A-438079 3 μg groups, respectively). BBG, Brilliant blue G; OxATP, adenosine 5′-triphosphate-2′,3′-dialdehyde; S.E.M., standard error of the mean.

Spatial memory was evaluated using the Morris water maze. During the five-day hidden platform trial, escape latency of all the five groups decreased in a day-dependent pattern. However, the sham group took significantly less time to find the platform than the saline group on all five days. In addition, the saline group required significantly more time to find the platform than the BBG 10 μg and A-438079 3 μg groups after the ninth day(+9D). The OxATP 1 μg group took a significantly longer time to find the platform than the A-438079 3 μg group after +9D and a shorter time than the saline group after the eleventh day (+11D). No significant differences existed among the sham, BBG 10 μg and A-438079 3 μg groups (Figure 3A), and no significant differences in swimming speed were observed among the five groups (data not shown).

In the probe trial, the saline group spent significantly less time than the other four groups in the SW quadrant. There was no significant difference among the sham, BBG 10 μg group and OxATP 1 μg and A-438079 3 μg groups (Figure 3B).

### Inhibition of P2X7Rs reduces I/R-induced glial activation

**Figure 4:**
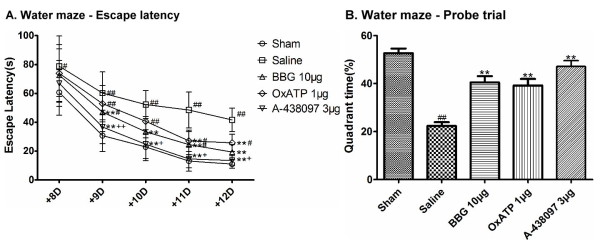
**Effect of BBG, OxATP and A-438079 on I/R-induced astroglial and microglial activation. (A)** Representative immunoreactivity for GFAP (left) and Iba-1 (right) in the hippocampal CA1 region at the third day (+3D). (**B)** Quantitative analysis of IOD values of GFAP in the hippocampal CA1 region at +3D. (**C**) Quantitative analysis of IOD values of Iba-1 in the hippocampal CA1 region at +3D. Values are expressed as the mean ± S.E.M. All photomicrographs are × 400. Scale bar: 10 μm. **P < 0.01 versus saline group; ##P < 0.01 versus sham group; #P < 0.05 versus sham group(n = 4 in all the groups). BBG, Brilliant blue G; GFAP, glial fibrillary acidic protein; IOD, integrated optical density; Iba1, ionized calcium-binding adaptor molecule 1; I/R, ischemia/reperfusion; OxATP, adenosine 5′-triphosphate-2′,3′-dialdehyde; S.E.M., standard error of the mean.

To investigate the association between P2X7Rs and ischemia-induced neuroinflammation, we evaluated microglial and astroglial activation at +3D using an immunohistochemistry method. Astrocytes were identified with an antibody against GFAP. In the sham group, only a few astrocytes with thin and long processes were stained positive. However, a robust increase in GFAP immunoreactivity and hypertrophic cellular morphology of astrocytes was observed in the saline group. Treatment with BBG 10 μg, OxATP 1 μg or A-438079 3 μg markedly attenuated the increase in GFAP immunoreactivity compared to the saline group (Figure 4A,B).

Iba-1 is a specific marker for microglia. Immunostaining for Iba-1 revealed that in the sham group, only a few scattered ramified microglia (resting microglia) were observed. After three days of reperfusion, the number of microglia was markedly increased in the hippocampal CA1 region, the resting microglia turned into amoeboid-like cells with plump cell bodies and short, thick processes which reflected morphological features of activated microglia. There was a significant decrease in microglial activation and infiltration in the BBG 10 μg, OxATP 1 μg and A-438079 3 μg groups when compared to the saline group (Figure 4A,C).

### Inhibition of P2X7Rs attenuated I/R-induced cytokine overexpression

**Figure 5:**
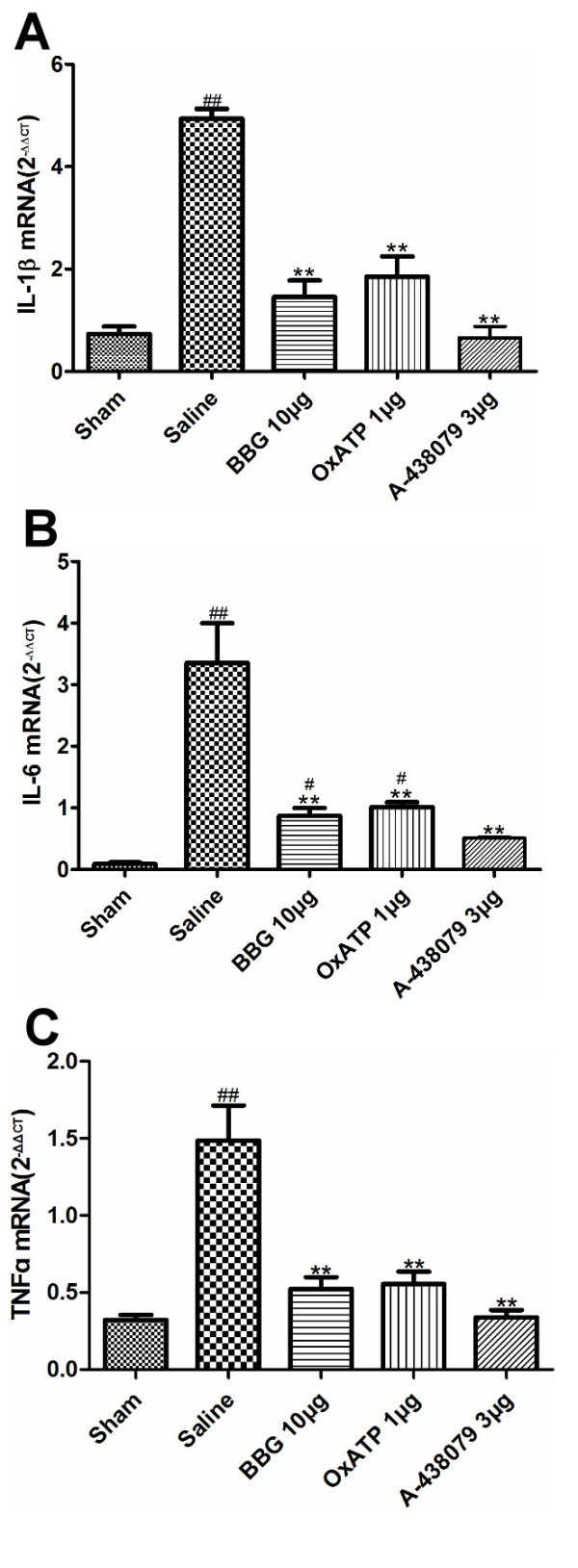
**Effect of BBG, OxATP and A-438079 on I/R -induced IL-1ß, TNF-α and IL-6 overexperession.** Steady-state mRNA expression for IL-1ß (**A**), TNF-α (**B**) and IL-6 (**C**) quantified by real-time RT-PCR. Expression is relative to GAPDH. Values are expressed as the mean ± S.E.M. **P < 0.01 versus saline group; ##*P* < 0.01 versus sham group (n = 7 in all the groups). BBG, Brilliant blue G; GAPHD, glyceraldehyde-3-phosphate dehydrogenase; IL-1ß, interleukin-1ß; IL-6, interleukin-6; I/R, ischemia/reperfusion; OxATP, adenosine 5′-triphosphate-2′,3′-dialdehyde; S.E.M., standard error of the mean; TNF-α, tumor necrosis factor α.

To determine the effect of inhibiting P2X7Rs on hippocampal inflammatory cytokine production, the expression levels of three cytokines, IL-1ß, TNF-α and IL-6 were tested by RT-PCR at +3D. As expected, transient global cerebral I/R significantly increased mRNA expression of IL-1ß, TNF-α and IL-6 in the hippocampus. Administration of BBG 10 μg, OxATP 1 μg or A-438079 3 μg markedly attenuated the I/R-induced overexpression of IL-1ß, TNF-α and IL-6 (Figure 5).

## Discussion

In this study, we demonstrated for the first time that inhibiting P2X7Rs protects against transient global cerebral I/R injury via modulating inflammatory responses in the rat hippocampus. When BBG and OxATP, two of the most widely used P2X7R antagonists, and A-438079, a selective P2X7R antagonist, were centrally administrated right before transient global cerebral I/R injury, they (1) reduced mortality, neuronal cell death and behavioral deficits, and (2) reduced the inflammatory responses as evidenced by a reduction in microglial and astroglial activation, and decreased inflammatory cytokine expression.

Cerebral ischemia rapidly increases inflammatory responses in the rodent brain, which is characterized by astroglial and microglial activation and inflammatory cytokine release [2,25-27]. Transient global cerebral I/R leads to selective tissue damage in the hippocampal CA1 region, and neuronal death in the CA1 region after global cerebral ischemia has occurred in a delayed manner [27,28]. In our present study, apparent neuronal death was observed in the hippocampal CA1 region in the saline group after three to seven days of reperfusion, accompanied by marked glial activation and cytokine overexpression (IL-1ß, TNF-α and IL-6). Astroglial and microglial activation in the hippocampus not only induces the production of inflammatory cytokines but also reactive oxygen species, chemokines, proteases, and vasoactive mediators many of which are cytotoxic to neuronal cells [29]. Taken together, our findings proved that neuroinflammation following transient global cerebral I/R injury is an important contributor to I/R-induced hippocampal CA1 neuron death.

The P2X7R is predominantly expressed by microglial cells in the CNS. Numerous literature reports have shown that P2X7R stimulation is related to microglial activation, high doses of ATP that elicit microglia proliferation [30,31] and morphological transformation [31], as well as superoxide production [32] and inflammatory cytokine secretion [33,34] which could be inhibited by P2X7R antagonists. Astrocytes normally express low levels of P2X7R [35-37]. However, the expression levels would be elevated in some pathological conditions [38,39], thus the astroglial P2X7R may be a direct target of ATP as an immunoregulator. Recently, Jae *et al*. [9] reported that BBG reduced the activation of astrocytes and microglia as well as neuronal death in the hippocampus of amyloid-ß_1–42_ (Aß_1–42_)-injected rats. Pengetal. [8] also found that BBG treatment could attenuate glial activation, tissue damage and behavioral deficits in a rat spinal cord injury model. In agreement with these studies, our data showed that BBG, OxATP and A-438079 reduced I/R-induced microglial and astroglial activation, which was accompanied by reduced hippocampal CA1 neuron death, reduced apoptosis, and better spatial memory recovery(Figure 5), These results suggest that P2X7 receptor-mediated microglial and astroglial activation plays a crucial role in neuronal damage in the hippocampal CA1 region following transient global cerebral I/R injury.

Reactive microglia and astrocytes can produce and release various inflammatory cytokines. Our results showed that inhibition of P2X7Rs reduced the expression levels of IL-1ß, TNF-α and IL-6 after transient global cerebral I/R injury. ATP is a potent inducer of IL-1ß release as it activates the P2X7R-caspase 1 pathway [40]. The P2X7R also mediates TNF-α and IL-6 secretion through diverse signal cascades [34,41]. In this study, transient global cerebral I/R injury resulted in a substantial increase in the expression levels of TNF-α, IL-1β, and IL-6 in the rat hippocampus. We noted that in the BBG 10 μg, OxATP 1 μg and A-438079 3 μg treated groups, the levels of pro-inflammatory cytokines were significantly suppressed, which suggests that inhibition of the P2X7R effectively suppresses I/R-induced surges in inflammatory cytokines in the hippocampus.

An increased number of P2X7R antagonists have been identified and optimized by pharmaceutical companies and academic groups. Among them, BBG and OxATP are the most widely used in recent studies of P2X7Rs [6]. However, both P2X7R antagonists suffer from relatively poor receptor specificity. OxATP has strong antagonistic effects not only on P2X7Rs, but also on other P2XRs [42,43], and even P2YRs. BBG has nanomolar affinity on rat P2X7 receptors and only micromolar affinity at some other P2XRs, and used to be the most selective antagonist prior to application of the selective P2X7R antagonist A-438079 in 2007 [43]. Our results show that all three P2X7R antagonists can protect hippocampal CA1 neurons from global cerebral I/R injury. Rats receiving A-438079 3 μg had higher neuron survival rates and better motor performance than those receiving OxATP 1 μg. This may be due to the complex mechanism of OxATP on other P2Rs, which has not been well studied in cerebral ischemia.

Previous studies of Le Feuvre*et al*. [17] and Yanagisawa *et al*. [16] failed to show any protective effect of OxATP in MCAO models. OxATP is an irreversible antagonist of P2X7Rs, which is widely used in studies of P2X7Rs. OxATP in high concentrations have been reported to be severely cytotoxic to cerebellar granule neurons [6,44] and macrophages [6]. Although an *in vivo* study [10] showed i.c.v. injection of high doses (30 μg/day) of OxATP is safe for rats, the concentration of the injected solution was relatively low (5 mM), and the speed of injection was very slow (1.2 μg/hr). In the study by Yanagisawa*et al*. [16], OxATP was injected (i.c.v) at a concentration of 100 mM (totally 101 μg in 2 μL), which is much higher than the OxATP concentration used in our study (1 mM, totally 1 μg in 2μL). OxATP at high doses and concentrations may be fatal to neuronal cells. Le Feuvre*et al*. [17] used 3 mMOxATP (totally 3 μg in 2 μL) in a mouse MCAO model, and did not observe any protective effect of OxATP. Based on the report of Hibell*et al*. [45], apparent species differences exist in the kinetic properties of P2X7R antagonists, thus the dosage used in Le Feuvre’s study may not be optimal for mice. However, further studies are needed to elucidate the pharmacokinetics of different P2X7R antagonists *in vivo*.

Yanagisawa’s study [16] has shown that 2′, 3′-O-(4-benzoyl-benzoyl)adenosine5′-triphosphate (BzATP), a potent P2X7R agonist protected against MCAO injury in rats. BzATP was widely used in studies of P2X7Rs, as it exhibited at least 10- to 30-fold greater potency than ATP [46]. However, BzATP was not a selective P2X7R agonist; it could also bind to other P2X receptors, especially P2X1R, P2X2R and P2X3R, and has much more potent effects on P2X1 and P2X3 receptors than P2X7Rs [6,47]. Therefore, the mechanisms of BzATP-induced neuroprotection in the rat brain may be far more complicated than activation of P2X7Rs. Unfortunately, to our knowledge, there are no available pharmacological ligands that can selectively activate the P2X7R [47]. Further research should be aimed at developing P2X7 agonists with greater pharmacologic specificity.

## Conclusions

Overall, our study has shown that neuroinflammation following transient global cerebral I/R injury is an important contributor to hippocampal CA1 neuron death, and that inhibition of P2X7Rs protects against transient global cerebral I/R injury by reducing I/R-induced inflammatory responses. These results indicate that blocking P2X7Rs may be a promising therapeutic strategy for clinical treatment of transient global cerebral I/R injury.

## Abbreviations

ANOVA: analysis of variance; BBG: Brilliant blue G; BzATP: 2′, 3′-O-(4-benzoyl-benzoyl)adenosine5′-triphosphate; CBF: cerebral blood flow; CCAs: common carotid arteries; CNS: central nervous system; GAPHD: glyceraldehyde-3-phosphate dehydrogenase; GFAP: glial fibrillary acidic protein; H & E: hematoxylin and eosin; Iba1: ionized calcium-binding adaptor molecule 1; i.c.v.: intracerebroventricular; IgG: immunoglobulin G; IL-1ß: interleukin-1ß; IL-6: interleukin-6; IOD: integrated optical density; I/R: ischemia/reperfusion; MCAO: middle cerebral artery occlusion; OxATP: adenosine 5′-triphosphate-2′,3′-dialdehyde; P2X7R: P2X7 receptor; PBS: phosphate-buffered saline; S.E.M.: standard error of the mean; TNF-α: tumor necrosis factor α; TUNEL: deoxynucleotidyl transferase-mediated UTP nick end labeling.

## Competing interests

The authors declare that they have no competing interests.

## Authors’ contributions

BL, QX and KC conceived the study. BL and QX participated in its design and coordination, KC, GP, JW and MF analyzed the data and drafted the manuscript. KC and BY participated in setting up the model of global cerebral I/R injury. KC, BY and YD performed behavioral testing on the rats. HL and ZX performed the immunohistochemistry and RT-PCR experiments. All authors have read and approved the final manuscript.
